# Cost-effectiveness analysis of Smart Triage, a data-driven pediatric sepsis triage platform in Eastern Uganda

**DOI:** 10.1186/s12913-023-09977-5

**Published:** 2023-08-31

**Authors:** Edmond C. K. Li, Abner Tagoola, Clare Komugisha, Annette Mary Nabweteme, Yashodani Pillay, J. Mark Ansermino, Asif R. Khowaja

**Affiliations:** 1 Department of Anesthesiology, Pharmacology & Therapeutics, University of British Columbia, Vancouver, BC Canada; 2 Department of Anesthesiology, Royal Columbian Hospital, Vancouver, BC Canada; 3 Jinja Regional Referral Hospital, Jinja, Uganda; 4 World Alliance for Lung and Intensive Care Medicine in Uganda, Kololo, Kampala, Uganda; 5 Center for International Child Health, British Columbia Children’s Hospital, Vancouver, BC Canada; 6 Faculty of Applied Health Sciences, Brock University, St. Catharines, ON Canada

**Keywords:** Cost-effectiveness analysis, Economic evaluation, Pediatric sepsis, Triage, Low-middle income country, Sepsis

## Abstract

**Background:**

Sepsis, characterized by organ dysfunction due to presumed or proven infection, has a case-fatality over 20% in severe cases in low-and-middle income countries. Early diagnosis and treatment have proven benefits, prompting our implementation of Smart Triage at Jinja Regional Referral Hospital in Uganda, a program that expedites treatment through a data-driven triage platform. We conducted a cost-effectiveness analysis of Smart Triage to explore its impact on patients and inform multicenter scale up.

**Methods:**

The parent clinical trial for Smart Triage was pre-post in design, using the proportion of children receiving sepsis treatment within one hour as the primary outcome, a measure linked to mortality benefit in existing literature. We used a decision-analytic model with Monte Carlo simulation to calculate the cost per year-of-life-lost (YLL) averted of Smart Triage from societal, government, and patient perspectives. Healthcare utilization and lost work for seven days post-discharge were translated into costs and productivity losses via secondary linkage data.

**Results:**

In 2021 United States dollars, Smart Triage requires an annuitized program cost of only $0.05 per child, but results in $15.32 saved per YLL averted. At a willingness-to-pay threshold of only $3 per YLL averted, well below published cost-effectiveness threshold estimates for Uganda, Smart Triage approaches 100% probability of cost-effectiveness over the baseline manual triage system. This cost-effectiveness was observed from societal, government, and patient perspectives. The cost-effectiveness observed was driven by a reduction in admission that, while explainable by an improved triage mechanism, may also be partially attributable to changes in healthcare utilization influenced by the coronavirus pandemic. However, Smart Triage remains cost-effective in sensitivity analyses introducing a penalty factor of up to 50% in the reduction in admission.

**Conclusion:**

Smart Triage’s ability to both save costs and avert YLLs indicates that patients benefit both economically and clinically, while its high probability of cost-effectiveness strongly supports multicenter scale up. Areas for further research include the incorporation of years lived with disability when sepsis disability weights in low-resource settings become available and analyzing budget impact during multicenter scale up.

**Trial registration:**

NCT04304235 (registered on 11/03/2020, clinicaltrials.gov).

**Supplementary Information:**

The online version contains supplementary material available at 10.1186/s12913-023-09977-5.

## Background

Sepsis is a syndrome characterized by an inflammatory state triggered by a presumed or proven infection that results in organ dysfunction and/or death [1]. In severe cases in low-and-middle income countries (LMICs), disease-specific mortality and case fatality ratios can exceed 20% [2, 3]. In Uganda, the burden of sepsis weighs heavily on the healthcare system and pediatric population, with a neonatal prevalence of 24.4% [4]. International sepsis treatment guidelines recommend delivering a bundle of care within one hour (defined in this paper as a “timely sepsis bundle”), based on evidence that earlier treatment reduces mortality and improves patient outcomes [5]. This bundle consists of antimicrobials alongside fluid and oxygen as clinically indicated [5].

In high income countries, evidence-based triage strategies aimed at expediting sepsis treatment [6] involve laboratory investigations often inaccessible in LMIC settings, where delays in treatment leading to a high case fatality ratio are commonplace [7]. LMIC-specific guidelines such as the Emergency Triage Assessment and Treatment (ETAT) [8] and others [9, 10], show good predictive power for mortality and improve clinical outcomes when successfully implemented [11–13]. However, the complexity of these guidelines face challenges in implementation, particularly in resource-poor environments where patient throughput and new-staff turnover is high [14, 15]. Infrastructural barriers and medication shortages further delay sepsis treatment even if a sick child has been effectively identified as having sepsis [15].

The Smart Triage program, which was implemented at the pediatric outpatient department at Jinja Regional Referral Hospital (JRRH) from 2020–2021, targeted these barriers in several ways [16]. First, we developed and implemented a model that used clinical variables for risk stratification based on the child’s predicted need for admission. This allowed sicker children to be prioritized during assessment by the existing JRRH clinical team [17]. This model was housed in a mobile application which required minimal training (compared to existing triage guidelines) for use by new team members. Secondly, a Bluetooth patient and treatment tracking system enhanced patient flow and organization by conveying patient status and risk stratification to the clinical team through an electronic dashboard in an automated manner. Thirdly, outcome data from this patient tracking system, such as time taken to deliver a sepsis bundle of care, was used to drive local quality improvement cycles. This combination of quality improvement cycles with a data-driven approach has been shown to effectively improve patient outcomes in the LMIC setting [18].

In high-income settings, economic evaluations of quality improvement initiatives aimed at delivering early sepsis care have demonstrated reduced cost and increased benefit over standard of care [19–21]. However, such analyses have not been comprehensively conducted for triage programs in LMICs. LMIC analyses that do exist may report partial costs only [14], or report intermediate outcomes such as quality improvement scores with limited clinical interpretability [22]. In addition, these analyses focus on the perspective of the healthcare system [13, 22, 23], but do not consider out-of-pocket costs and productivity losses. In LMICs such as Uganda that are yet to implement universal health coverage (UHC), where patients may incur out-of-pocket costs near or exceeding that of monthly earnings [24], inclusion of the societal perspective is important to explore the economic impact of healthcare interventions on patients. Lastly, no LMIC analysis has utilized economic modelling techniques to incorporate healthcare utilization costs and link intermediate outcomes to clinical endpoints. To address these gaps in the literature, we conducted a cost-effectiveness of the Smart Triage program using a decision analytic model from a societal perspective, using secondary linkage data to link healthcare utilization to costs, lost work to productibility losses, and process outcomes to clinical endpoints. Results of this analysis may be used both to explore the impact of Smart Triage on patients and to inform scale up of Smart Triage to other Ugandan centers.

## Methods

### Objectives

1. Determine the incremental cost effectiveness ratio (ICER) for years-of-life lost (YLL) averted by the Smart Triage program compared to the baseline triage infrastructure at JRRH, using the pre-intervention phase at JRRH as the control.
2. Determine the probability of cost-effectiveness of the Smart Triage program under different willingness-to-pay thresholds, compared to the baseline triage infrastructure at JRRH.
3. Investigate the impact of out-of-pocket costs on cost-effectiveness by secondary analyses separating the government and patient perspectives.

### Description of primary clinical trial

The Smart Triage program, and corresponding clinical trial, were implemented in the pediatric outpatient department at JRRH in Uganda, which serves children up to age 18 years presenting at various degrees of illness severity. A concurrent control site where Smart Triage would not be implemented was originally planned, but coronavirus disease (COVID) pandemic-induced delays in site initiation precluded the use of this site as a control for our analysis. Therefore, the clinical trial, and data used for this analysis, is of a pre-post design. The comparator to Smart Triage was a manual triage system used in the pre-implementation phase at JRRH that is based on ETAT, a set up common in many comparable LMIC centers [25]. The pre-intervention phase stretched from April 2020 to December 2020, while the post-intervention phase stretched from April 2021 to December 2021. January through March 2021 corresponded to the bulk of program inception/development, and outcome data from these months were not included in our analysis.

All presenting patients, except for those with scheduled appointments or procedures, were eligible for enrollment. Primary outcome was defined as the difference, between the pre- and post-implementation phases, in the proportion of children who received a timely sepsis bundle (defined as within one hour of arrival at the outpatient department). This choice of outcome is based on benchmarks established by international guidelines [1], and improving this outcome is associated with decreased mortality [26]. Enrolled children were followed up via telephone to their caregiver for in-hospital mortality (if admitted), 7-day post-discharge mortality, and 7-day post-discharge healthcare utilization rates. We optimized our follow-up rate through rigorous checks for phone number accuracy, repeat calls to unreached caregivers through a standardized process, and use of shared phone numbers, such as that of a neighbor, should a caregiver not have a personal number. This scheme has achieved a greater than 95% follow-up rate in previous studies by our group in the same region [27]. Further details on the Smart Triage program, clinical trial protocol, sample size calculations, and telephone follow-up have been published elsewhere [16] with appropriate clinical trial registration on clinicaltrials.gov (NCT04304235, 11/03/2020). Ethics approval was obtained from Makerere University School of Public Health (MUSPH) Higher Degrees, Research and Ethics Committee (protocol number 743). MUSPH provided approval for the parent study and this economic evaluation to be conducted at Jinja Regional Referral Hospital in Jinja, Uganda; all study procedures were performed in accordance with the Declaration of Helsinki.

### Economic model structure and assumptions

**Fig. 1:**
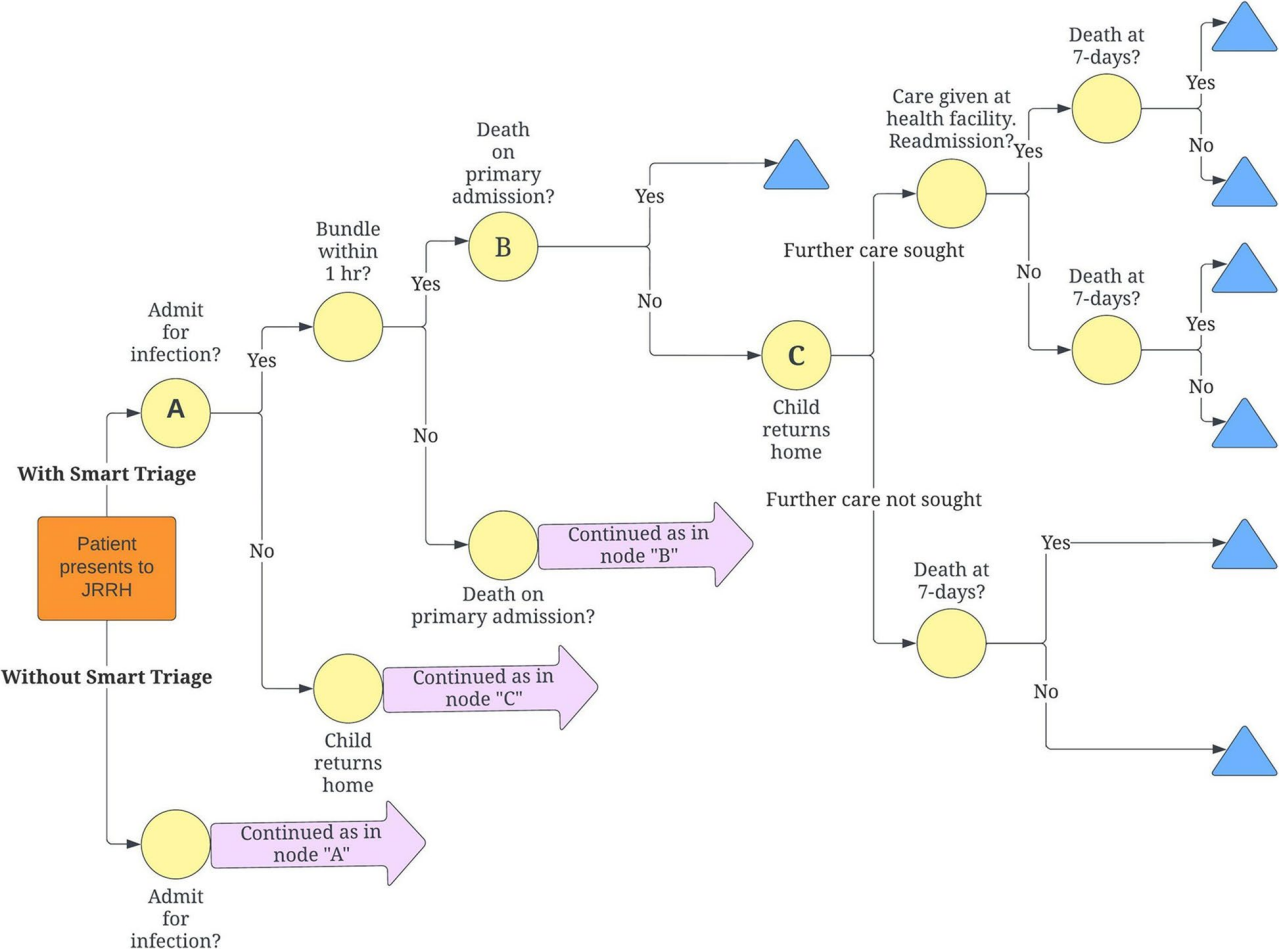
Decision analytic model for patient care pathway before and after Smart Triage implementation. USD = United States dollars, HOC = Health opportunity cost, WHO = World Health Organization, GDP = Gross domestic product

We conducted our analysis using a decision analytic model from a societal perspective, using secondary data from the literature to link health resource utilization (HRU) rates to costs and process outcomes to clinical endpoints [26, 28]. Sub-analyses were done from the government perspective to inform policy formation, and from the patient perspective to isolate economic impact on patients. The model (Fig. 1) mirrors a patient’s clinical pathway from the time of presentation at JRRH to the post-discharge period up to seven days. The structure of this model were validated in consultation with local clinicians at JRRH, and the post-discharge components mirror that of a recently conducted cost-effectiveness analysis of a post-discharge follow-up program for sepsis in Uganda [29]. Lastly, Smart Triage was implemented with heavy advocacy and leadership by local stakeholders for sustainability beyond the trial end date. Therefore, we assumed a program duration of five years for annuitization of program costs and calculation of total patients impacted.

Key assumptions of our model include the sustainability of Smart Triage beyond study end date, the generalizability of secondary linkage data used in the model to the Smart Triage population, and the adequacy of our seven-day duration of patient follow-up. The rationale behind these assumptions, and further details on our model, are previously published [30]. Our analysis was designed and conducted according to the 2013 version of the Consolidated Health Economic Evaluation Reporting Standards (CHEERS) statement [31], the most recent version at the time of analysis design, and is also in compliance with the revised 2022 version [32].

### Model inputs

As the Smart Triage trial was powered for the pre-post difference in proportion of children receiving a timely sepsis bundle and not mortality difference, the estimated improvement in the latter for any observed improvement in the former was calculated through the literature odds ratio for mortality improvement if given a timely sepsis bundle vs. not [26]. YLLs were then calculated via the product of: 1) mortality rate, 2) total number of patients, 3) difference between the average life expectancy in Uganda [33] and the average age of death in the Smart Triage trial. The pre-post difference in YLL represents the YLL-averted.

In terms of HRU costs, unit costs of care center visits with and without admission were obtained from the Decade of Vaccine Economics project [28, 34]. This source provided aggregate Ugandan HRU costing data from 2018 in pediatric pneumonia, a leading cause of sepsis in LMICs [35]. Costs were segregated by government vs. patient perspectives and outpatient visits vs. inpatient stays. Government costs included facility and medical costs attributable to a clinical visit not directly borne by the patient. For patient costs, both direct costs (e.g. hospital fees) and indirect costs excluding productivity losses (e.g. transportation) were included. Over 45 healthcare centers across Uganda were surveyed. The types and frequency of HRU observed in the Smart Triage trial were then multiplied with the relevant unit cost to obtain costing input for our analysis. For example, if a patient was admitted to JRRH for five days, the unit cost per diem for admission from the government perspective would be multiplied by five to obtain the corresponding cost on the system for admission. The same calculation would be repeated from the patient perspective to obtain out-of-pocket costs. To track productivity losses, we measured caregivers’ lost days of work directly from our trial, then multiplied by the average daily wage in Uganda from the International Labor Organization [36] to obtain lost wages. We counted as caregivers: parents, relatives, and non-family caregivers (e.g. neighbor).

Lastly, program costs were tabulated directly from our trial; costs solely attributable to research were excluded. Program costs were counted from a public payer (i.e., the government’s) perspective, as patients were not charged out-of-pocket fees for Smart Triage due to the reusable nature of program components. All costs were inflated to 2021, the implementation year of Smart Triage, via the gross domestic product (GDP) deflator of the country in which they incurred, then converted to United States dollar (USD) using 2021 average exchange rates. 2021 was chosen as that was the implementation year of Smart Triage.

### Perspectives of analysis

Our analysis from the government perspective includes costs of care not borne out-of-pocket by patients (e.g. healthcare worker salary, hospital utilities, Smart Triage program costs). Our analysis from the patient perspective includes out-of-pocket costs directly related to healthcare (e.g. hospital fees), costs indirectly related to having a hospitalized child (e.g. transportation), and productivity losses (lost wages). Our analysis from the societal perspective combines these two perspectives to create a wholistic picture of Smart Triage’s cost-effectiveness.

### Statistical analyses

Bivariate analyses were performed using the chi squared test for discrete outcomes and student t test for continuous outcomes. Fischer exact test was used in place of chi-squared test when the number of events were less than five. The incremental cost-effectiveness ratio (ICER) is represented by: (post–pre difference in cost)/(post–pre difference in YLL). The ICER was calculated by multiplying out the decision analytic model after filling in probabilities for each branch point, and summing the corresponding total costs and YLL for each terminal node [37]. Monte Carlo simulations with 10,000 iterations were used to simulate uncertainty of the ICER. Each of the 10,000 simulated ICERs were then plotted on a cost-effectiveness plane, with the vertical axis as costs and horizontal axis as YLL-averted. On this plane, simulations in the northeast quadrant represent increased clinical efficacy at an added cost, in the northwest quadrant reduced clinical efficacy at an added cost (should not adopt intervention), in the southwest quadrant reduced clinical efficacy but with cost savings, and in the southeast quadrant increased clinical efficacy with cost savings (economic dominance – adopt intervention). The proportion of simulations falling under each of these scenarios were calculated.

Next, the probability of cost-effectiveness under varying thresholds for willingness to pay per YLL averted were plotted on a cost-effectiveness acceptability curve (CEAC). The CEAC was generated by modelling increasing monetary amounts a payer would be willing to pay to avert one YLL; these amounts correspond to lines with increasing slopes on the cost-effectiveness plane. The proportion of simulated ICERs that fall below each line on the cost-effectiveness plane corresponds to the probability of cost-effectiveness at that willingness to pay threshold [37]. Therefore, plotting “proportion of ICERs below the line” on the vertical axis and “slope of line” on the horizontal axis creates the CEAC.

For the above analyses, annuitization and discounting rates of 3% per annum were used for costs and YLL, in congruence with a systematic review of other LMIC economic evaluations [38], assuming a five year program duration. All analyses were performed using R [39]. Additional details regarding statistical analyses and the Monte Carlo simulation may be found in our published protocol [30].

### Definition of cost-effectiveness

As mentioned above, the CEAC plots the probability of cost-effectiveness of Smart Triage over the baseline triage infrastructure at increasing willingness to pay thresholds. A cost-effectiveness threshold of a country is its chosen willingness to pay threshold to adopt an intervention if, at or below that threshold, an intervention has a high probability of cost-effectiveness over the comparator. However, most LMICs, including Uganda, do not have an formally established cost-effectiveness threshold. Therefore, for our analysis, we selected a threshold based on projected health opportunity costs (HOC) of Uganda (threshold = $174.12 in 2021 USD). As many cost-effectiveness analyses have historically used multiples of GDP per capita ($883.89 in 2021 USD for Uganda), as recommended by the World Health Organization (WHO), we have also stated our results in the context of this standard [40]. See “Discussion – strengths of analysis” section for our rationale for adopting the HOC-based threshold, along with limitations for using either threshold.

### Sensitivity analyses

We performed one-way deterministic sensitivity analyses using different annuitization rates (0%, 1%, 5%), and by removing the assumption that Smart Triage would continue past the study end date. The upper limit of 5% was chosen in congruence with variations in economic evaluation guidelines in global health [41], stemming from the variations in economic growth rates of different LMICs [42]. In a scenario-based sensitivity analysis, we also excluded months where patient load at JRRH outpatient department was lower than 50% of the annual median, to account for months where patient load abnormally slowed down due to COVID-pandemic induced travel restrictions. The rationale for this sensitivity analysis was that the primary outcome of Smart Triage, receiving a timely sepsis bundle, is a process outcome that may be affected by decreases in patient load from reasons such as travel restrictions. For this sensitivity analysis, the months excluded were September 2020 from the pre-intervention phase; and January, June, July, August 2021 from the post-intervention phase; corresponding to months where patient load reduced to lower than 50% of the annual median. For each one-way sensitivity analysis, we also applied a probabilistic sensitivity analysis using Monte Carlo simulation to account for parameter uncertainties within our model, as described previously.

Since the cost-effectiveness of Smart Triage was driven mainly by a reduction in admissions rather than an improvement in receiving a timely sepsis bundle, we performed a post-hoc sensitivity analysis whereby the absolute reduction in admission was further reduced in magnitude by 25%, 50%, and 75%. The rationale for this sensitivity analysis is that a portion of the reduction in admission rate may be driven by other factors independent from the efficacy of Smart Triage, including changing HRU behaviors throughout the COVID pandemic.

## Results

### Demographics and conduct of Smart Triage trial

The sex and age distribution of children were similar between the pre- and post-intervention phases, with the vast majority (over 85%) being children under 5 years. Malnutrition rates and HIV positivity were evenly distributed between phases. The rate of HIV positivity was low, but more than a third of patients have never been tested. Among admitted patients, greater than 90% were admitted for suspected infection. Diagnoses for non-admitted patients were not gathered, due to constraints in trial human resources. Approximately 10% of patients received a triage category of “emergency”, while the remaining 90% were split between “priority” and “non-urgent.” Of note, 41 patients were missing triage priority data due to technical difficulties. Data recovery was unfortunately not possible despite the best efforts of the research team. However, triage priority was not an input variable to the economic model and therefore does not affect our analysis and key findings. Lastly, greater than 90% of patients resided outside of the Jinja Central municipality, thereby necessitating some form of transportation to JRRH. We achieved a high rate of follow-up, with only 1.8% and 1.4% of patients lost to follow-up in the two phases respectively (Table 1).

**Table 1:** Demographics of patients enrolled into the Smart Triage trial at Jinja Regional Referral Hospital

Variables	Pre-intervention phase (%/sd)	Post intervention phase (%/sd)
Dates corresponding to each phase	April 27, 2020 to December 31, 2020	April 1, 2021 to December 31, 2021
Number of enrolled patients	1402	1942
Male to female	729 (52.0) to 673 (48.0)	1015 (52.3) to 927 (47.7)
Mean age (years)	2 (2.5)	2 (2.0)
Age < 5 years	1246 (88.9)	1794 (92.4)
Wasting (acute malnutrition)		
Moderate (height to weight Z score -2 to -3)	76 (5.4)	62 (3.2)
Severe (height to age Z weight < -3)	79 (5.6)	46 (2.4)
Stunting (chronic malnutrition)		
Moderate (height to age Z score -2 to -3)	166 (11.8)	189 (9.7)
Severe (height to age Z score < -3)	131 (9.3)	167 (8.6)
HIV status		
Positive	8 (0.6)	14 (0.7)
Negative	900 (64.2)	1216 (62.6)
Unknown	494 (35.2)	671 (34.6)
Hospital admission within last 6 months	268 (19.1)	285 (14.7)
Infection admission diagnosis	297 (91.7)	281 (96.2)
Triage priority by Smart Triage platform		
Non-urgent	NA	857 (44.1)
Priority	NA	840 (43.3)
Emergency	NA	204 (10.5)
Travelled from outside Jinja central (where JRRH is located)	1285 (91.7)	1766 (90.9)
Lost to follow-up	25 (1.8)	27 (1.4)
No phone number	6 (0.4)	5 (0.3)
Wrong phone number recorded	11 (0.8)	18 (0.9)
No answer on follow-up	8 (0.6)	4 (0.2)

NB: The pre-intervention phase was shorter by a month due to a delayed start at the study center for pandemic-related logistical reasons

Count data shown as count (%), continuous data shown as mean (sd)

*sd* standard deviation, *JRRH* Jinja Regional Referral Hospital, *HIV* human immunodeficiency virus

### Clinical outcomes

The parent clinical trial did not find statistically significant differences between the pre and post-implementation phases in the proportion of children receiving a timely sepsis bundle, in-hospital mortality, or 7-day post-discharge mortality. However, for total mortality, there was a statistically significant improvement in the post-intervention phase (absolute risk reduction = -0.6% [95% CI -1.2 to -0.05, *p* = 0.016]). In the pre-implementation phase, 0.7% (95% CI 0.4 to 1.3) of children received a timely sepsis bundle, compared with 0.7% (95% CI 0.4 to 1.2) of children in the post-implementation phase, (*p* > 0.99). Among admitted patients, we found in-hospital mortality rates of 2.9% (95% 1.5 to 5.5) and 1.1% (95% CI 0.22 to 3.4) during the pre- and post-implementation phases respectively (*p* = 0.13). Finally, follow-up completed 7 days after return home showed a 7-day mortality rate of 0.2% (95% CI 0.0 to 0.6) and 0.1% (95% CI 0.0 to 0.4) in the pre- and post-implementation phases respectively (*p* = 0.798) (Table 2). The average age of death during the Smart Triage trial, used to calculate YLL averted in the Monte Carlo simulation, was 2.0 years (95% CI 0.7 to 3.0).

**Table 2:** Clinical outcomes in the pre-implementation and post-implementation phases of Smart Triage

	**Pre-intervention phase (95% CI) (*N* = 1402)**	**Post-intervention phase (95% CI) (*N* = 1942)**	***p*-value**	**RR (95% CI) if statistically significant**	**ARR (95% CI) if statistically significant**
**Received sepsis bundle within one hour (%)**	0.7 (0.4 to 1.3)	0.7 (0.4 to 1.2)	> 0.99	NS	NS
**In-hospital mortality for admitted patients (%)**	2.9 (1.5 to 5.5)	1.1 (0.22 to 3.4)	0.13	NS	NS
**7-day mortality after leaving JRRH (%)**	0.2 (0.0 to 0.6)	0.1 (0.0 to 0.4)	0.798*	NS	NS
**Total mortality (%)**	0.9 (0.5 to 1.5)	0.3 (0.1 to 0.7)	0.016	0.33 (0.13 to 0.89)	-0.6 (-1.2 to -0.05)

*JRRH* Jinja Regional Referral Hospital, *RR* relative risk, *ARR* absolute risk reduction, *NS* not significant

Fisher exact test used instead of Chi-squared test where indicated (*)

### Program costs

The cost of implementing the Smart Triage platform, annuitized over a five-year duration, was $1369.82 per year in 2021 USD. With an average of 28,000 children presenting at JRRH’s outpatient department per year from 2020 to 2022, the average program cost per child is $0.05. Program costs were comprised of infrastructural costs (e.g. electronic dashboard indicating patient triage status), shared consumables (e.g. reusable radiofrequency patient tracking identification bands), or wages (e.g. one-time technologist fee for set-up). Details are available in Additional file 1.

### Health resource utilization

There was a clinically and statistically significant reduction in the admission rate at JRRH between the pre-intervention and post-intervention phases (-8.4% [95% CI -11.1 to -5.7], *p* < 0.001). There were also reductions in seeking care (-2.8% [95% CI -4.5 to -1.1], *p* = 0.001) and readmissions at any health facilities (-1.2% [95% CI -2.1 to -0.3], *p* = 0.008) after leaving JRRH. The mean length of stay for primary admission and readmission for both phases were 4 days (Table 3).

**Table 3:** Health resource utilization probabilities in the pre-implementation and post-implementation phases

	**Pre-intervention phase (95% CI) (*N* = 1402)**	**Post-intervention phase (95% CI) (*N* = 1942)**	***p*-value**	**RR (95% CI) if statistically significant**	**ARR (95% CI) if statistically significant**
**Rate of admission (%)**	22.3 (20.2 to 24.6)	13.9 (12.4 to 15.5)	< 0.001	0.62 (0.54 to 0.72)	-8.4 (-11.1 to -5.7)
**Rate of seeking any care after leaving JRRH (%)**	7.9 (6.6 to 9.4)	5.1 (4.2 to 6.2)	0.001	0.65 (0.50 to 0.84)	-2.8 (-4.5 to -1.1)
**Rate of readmission after leaving JRRH (%)**	2.4 (1.7 to 3.4)	1.2 (0.8 to 1.8)	0.008	0.50 (0.30 to 0.84)	-1.2 (-2.1 to -0.3)
**Mean length of stay if admitted at JRRH (days)**	4 (3.8 to 4.5)	4 (3.9 to 4.4)	0.646	NS	NS
**Mean length of stay for readmission (days)**	4 (2.8 to 4.8)	4 (3.1 to 5.1)	0.312	NS	NS

*JRRH* Jinja Regional Referral hospital, *RR* relative risk, *ARR* absolute risk reduction, *NS* not significant

For each pattern of HRU (admit vs. non-admit at JRRH and readmission vs. seek care again without readmission vs. no revisit at all after leaving JRRH), average days of work missed are shown in Table 4, along with corresponding losses in productivity. As expected, HRU patterns that involve admission and/or readmission results in greater missed work, and therefore higher productivity losses.

**Table 4:** Average days of paid employment missed by caregivers, stratified by health resource utilization pattern

	**Average days of paid employment missed (95% CI)**	**Productivity cost (2021 USD) (95% CI)**
**Admitted at JRRH, no further care sought**	4 (4 to 5)	13.59 (11.32 to 15.53)
**Not admitted at JRRH, no further care sought**	2 (2 to 2)	6.15 (5.50 to 6.79)
**Admitted at JRRH, readmitted at any center after discharge**	12 (5 to 18)	39.79 (17.15 to 56.62)
**Not admitted at JRRH, admitted at any center after leaving JRRH**	6 (5 to 7)	18.44 (14.56 to 22.00)
**Admitted at JRRH, sought further care after discharge without readmission**	6 (3 to 8)	17.79 (9.38 to 25.24)
**Not admitted at JRRH, sought further care after leaving JRRH without readmission**	4 (3 to 4)	12.29 (10.35 to 13.91)

*JRRH* Jinja Regional Referral hospital, *USD* United States dollar

### Cost effectiveness of Smart Triage – societal perspective (base-case analysis)

**Fig. 2:**
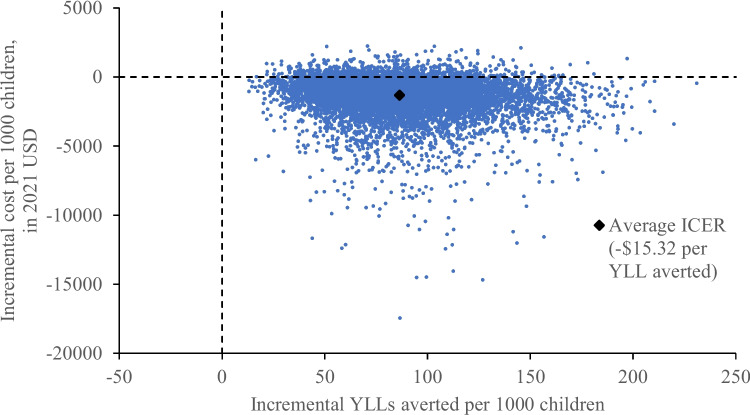
Cost effectiveness of Smart Triage relative to the baseline triage infrastructure at JRRH, societal perspective. USD = United States dollars, YLL averted = years of life lost averted, JRRH = Jinja Regional Referral Hospital

**Fig. 3:**
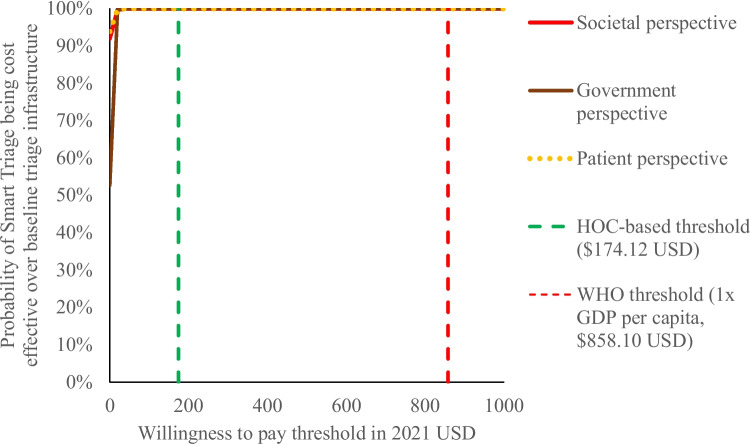
Cost effectiveness acceptability curve of Smart Triage relative to the baseline triage infrastructure at JRRH. USD = United States dollars, HOC = Health opportunity cost, WHO = World Health Organization, GDP = Gross domestic product

From the societal perspective, Smart Triage resulted in an incremental cost of -$1321.86 per 1000 children (95% CI -4735.48 to 444.73) and an incremental YLL averted of 86.3 years per 1000 children (95% CI 37.7 to 152.3), corresponding to an average ICER of -$15.32 per YLL averted ($15.32 saved per YLL averted). On the cost-effectiveness plane, 91.9% of the Monte Carlo simulations fall within the southeast quadrant, corresponding to both cost-savings and improved clinical outcome, while the remaining 8.1% of simulations fall within the northeast quadrant, corresponding to improved clinical outcome at an added cost (Fig. 2, Additional file 2). On the CEAC, 95% probability of cost-effectiveness was reached at approximately $3 USD, well before both the HOC-based threshold of $174.12 USD and WHO threshold for an “extremely cost-effective intervention” of 1 × GDP per capita ($883.89 USD) (Fig. 3).

### Government and patient perspectives

**Fig. 4:**
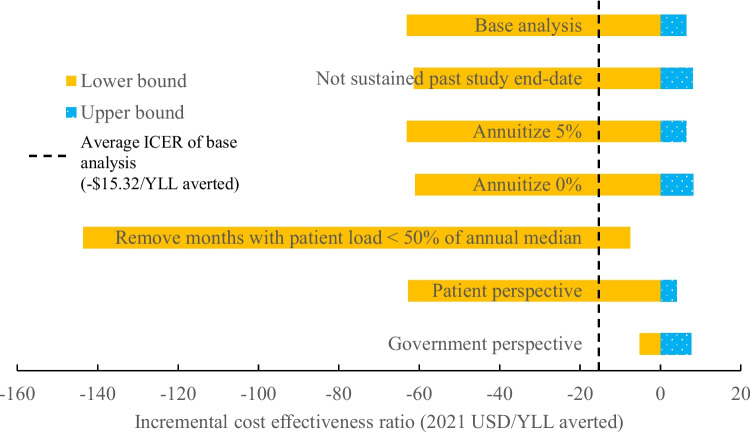
Tornado diagram showing variations in 95% confidence interval for ICER for various one-way sensitivity analyses. ICER = Incremental cost effectiveness ratio, YLL averted = Years of life lost averted, USD = United States dollars

From the government perspective, Smart Triage resulted in an incremental cost of $5.34 per 1000 children (95% CI -442.37 to 554.87) and an incremental YLL averted of 86.3 years per 1000 children (95% CI 37.2 to 153.1), corresponding to an average ICER of $0.06 per YLL averted. 52.7% of the Monte Carlo simulations result in both cost-savings and improved clinical outcome, while the remaining 47.3% result in improved clinical outcome at an added cost (Additional file 2). The ICER was less cost-effective compared to base case (Fig. 4), but 95% probability of cost-effectiveness was nonetheless reached well before both HOC-based and WHO thresholds (Fig. 3).

From the patient perspective, Smart Triage resulted in an incremental cost of -$1319.33 per 1000 children (95% CI -4604.14 to 319.00) and a YLL averted of 86.4 years per 1000 children (37.0 to 151.8), corresponding to an average ICER of -$15.27 per YLL averted ($15.27 saved per YLL averted). 93.9% of the Monte Carlo simulations result in both cost-savings and improved clinical outcome, while the remaining 6.1% result in improved clinical outcome at an added cost (Additional file 2). The ICER distribution was comparable to base case (Fig. 4), and 95% probability of cost-effectiveness was reached well before both HOC-based and WHO thresholds (Fig. 3).

### Sensitivity analyses 

The cost-effectiveness of Smart Triage was preserved in sensitivity analyses assuming annuitization rates of 0%, 1%, or 5%. Namely, 89.0%, 92.0%, and 91.9% respectively of the Monte Carlo simulations resulted in both cost-savings and improved clinical outcomes, while the remaining 11.0%, 8.0%, and 8.1% respectively resulted in improved clinical outcomes at an added cost (Additional file 3). ICERs showed similar distributions to the base analysis (Fig. 4). For all three scenarios, the probability of Smart Triage being cost-effective reached 95% by $4 USD, well under the HOC-based and WHO thresholds.

If we remove the assumption that Smart Triage would be sustained after study end date for a program duration of five years, cost-effectiveness was still maintained. In this scenario, only costs incurred/saved and YLL averted during the study timeframe were counted, and program costs were not annuitized. 89.2% of the Monte Carlo simulations resulted in both cost-savings and improved clinical outcomes, while 10.8% resulted in improved clinical outcomes with an added cost (Additional file 3). The ICER showed a similar distribution to the base analysis (Fig. 4). The probability of Smart Triage being cost-effective reaches 95% at $4 USD, well under the HOC-based and WHO thresholds.

Removal of months where patient attendance at the JRRH outpatient department dropped to 50% of the annual median showed an increase in cost savings and a corresponding shift towards added cost-effectiveness of the ICER compared to the base analysis. However, the confidence interval for cost and ICER were much wider than that of the base analysis (Fig. 4).

**Fig. 5:**
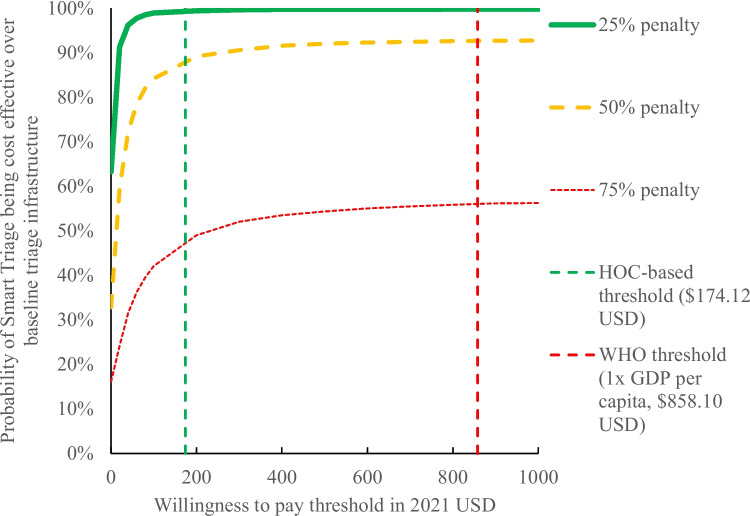
Cost effectiveness acceptability curve of Smart Triage, applying different penalty factors for admission rate reduction. USD = United States dollars, HOC = Health opportunity cost, WHO = World Health Organization, GDP = Gross domestic product

Our post-hoc analysis of reducing the absolute reduction in admissions by a penalty factor of 25%, 50%, and 75% of the observed value showed that a high probability of cost-effectiveness was attained below both HOC-based and WHO thresholds with a penalty factor of up to 50% (Fig. 5).

## Discussion

### Interpretation and impact of analysis

Our analysis shows Smart Triage achieves greater than 95% cost-effectiveness under a willingness to pay threshold calculated based on Uganda’s HOC (see “Discussion—strengths of analysis” section for further discussion). Furthermore, this cost-effectiveness is achieved well under the WHO threshold for defining an intervention as “extremely cost-effective” [43], which is one GDP per capita of the country of implementation ($858.10 in 2021 USD for Uganda) and is much higher than the HOC-based threshold of $174.12. For our base case analysis from the societal perspective, Smart Triage results in $15.32 USD saved per YLL averted (-$1321.86 [95%CI -4735.48 to 444.73] for 86.3 YLL averted [95%CI 37.7 to 152.3] per 1000 children), with a program cost of $0.05 USD per child. This small initial investment is only a minute fraction of the existing average hospital spending of $7 to $12 per child in Uganda for pneumonia, one of the most common causes of sepsis in LMICs [34]. Cost-effectiveness was not sensitive to changes in annuitization rate up to 5%. In addition, even if Smart Triage were not sustainable past the study end date, with incurred costs concentrated into the study period and no further YLL averted past the study period, cost-effectiveness persists.

We conducted our base case analysis from the societal perspective, with a sub-analysis from the patient perspective, to capture the economic impact of Smart Triage on patients, not just on the healthcare system like previous analyses [13, 22]. The patient perspective is important as a significant portion of healthcare costs are borne out of pocket in Uganda, which does not yet have UHC. In a Ugandan cross-sectional survey of pediatric sepsis patients, the hospital fee and medications alone cost $15 USD, increasing to $59 USD in private not-for-profit facilities [24]. In context, the average monthly wage in Uganda is $57 USD [36]. Additional costs not directly related to healthcare, such as transportation, meals while hospitalized, and hired childcare for siblings further increase the already heavy financial burden of having a hospitalized child [24, 44]. In this context, our analysis reassuringly indicates a high probability of economic dominance of Smart Triage from both the societal and patient perspectives (91.9% and 93.9%), suggesting that Smart Triage results in both cost-savings for patients and benefits in YLLs-averted, benefiting patients both economically and clinically.

From the government perspective, not only did the probability of cost-effectiveness approach 100% well before the HOC-based threshold (Fig. 3), but half of the Monte Carlo simulations were economically dominant over the pre-existing standard (Additional file 2), suggesting both cost savings and YLLs averted. A cross-sectional survey of Ugandan policy stakeholders showed that 95% felt that cost-effectiveness was an important factor in policy formulation, but unfortunately, a lack of personnel trained in economic evaluation coupled with limitations in information technology and data availability often prevent the use of cost-effectiveness analyses in drafting policy [45]. To this end, a favorable cost-effectiveness result for Smart Triage at JRRH provides strong economic rationale for further testing in additional centers to explore the adoption of Smart Triage into policy.

To assist in scaling up Smart Triage, our low program cost per child may be used to project initial investment cost at subsequent sites based on patient capacity, while scale up costs not encountered in our single center study may be recorded during scale up and used to model the budget impact of a wider adoption of Smart Triage in Uganda [46]. Additionally, implementation research on other successfully adopted health programs in sub-Saharan Africa show that a well-delineated scalable unit is paramount to successful expansion from a single test site to subsequent sites [47]. To this end, Smart Triage is designed to be implemented in an entire outpatient department, representing a clearly defined scalable unit. To further facilitate cost-effective scale up, we have minimized program costs by using non-proprietary and locally-acquired equipment where possible. Also, the training of local Smart Triage “champions” who would subsequently train new staff facilitated local familiarly with the platform that was sustained by existing local leadership rather than costlier extended support from high income country partners. These techniques to minimize capital cost and maximize local ownership are proven strategies towards sustainability in other newly scaled healthcare interventions in sub-Saharan Africa [27, 48, 49], and will be used during Smart Triage’s scale up.

Our analysis provides an evidence-based model and input parameters that could advance health economics research in the areas of pediatric sepsis care in Uganda and elsewhere. Our study findings also present policy implications and further research needs for the system-wide strengthening of pediatric sepsis care in Uganda. To this end,we are working with our partners at Walimu and the United Catholic Medical Bureau to expand the Smart Triage platform to four additional hospitals in Uganda. At these sites, we will conduct external validation of the model and solidify the platform’s scalability for further dissemination [50].

### Strengths of analysis

Our use of economic modelling techniques enabled the calculation of costs from healthcare utilization rates and linkage of intermediate outcomes (timely sepsis bundle) to clinical outcomes (YLL-averted). This technique results in a more comprehensive cost-effectiveness estimate to inform policy and allows for an estimate involving clinical endpoints such as YLL-averted despite our parent trial being powered for timely sepsis bundle, a process outcome. A previous economic evaluation of an ETAT-based pediatric triage system in Kenya, the ETAT + , also showed cost-effectiveness. However, this analysis utilized only program costs and the primary outcome of the parent cluster randomized trial [22]. As this primary outcome was a multi-domain quality improvement score, the ICER was difficult to interpret clinically. Another trial examining the ETAT + in Rwanda reported partial costs, but did not conduct a full cost-effectiveness analysis [14]. One analysis utilizing decision analytic modelling showed cost-effectiveness for the integrated management of neonatal childhood illness program in India, a program with some parallels to ETAT [51]. However, the interventions included encapsulated the entire inpatient and post-discharge timeline, with only a small fraction focusing on triage. To our knowledge, our use of economic modelling to evaluate a pediatric sepsis triage program is novel in the LMIC setting.

Another strength of our analysis is our novel use of an HOC-based willingness to pay threshold to define cost-effectiveness. Historically, cost-effectiveness analyses have used multiples of GDP per capita as defined by the WHO. While easy to understand, use of GDP per capita does not take into account an intervention’s cost-effectiveness relative to other existing interventions in a country [40, 52]. However, as all healthcare budgets are finite, funding a new intervention will by definition result in not funding other proposed interventions and/or cutting back on existing interventions. The HOC of a given country is defined as the current impact, per currency unit spent on existing healthcare interventions, on the disability adjusted life years (DALYs) averted in that country. Therefore, basing a cost-effectiveness threshold on HOC assesses novel interventions in the context of existing programs and allows for optimum allocation of resources in resource limited settings. The exact methodology to determine this threshold uses instrumental variables to account for hidden confounders and reverse causation between currency spent and DALYs averted. These methods are aptly described in the reference publication [53]. The HOC-based threshold for Uganda is $174.12 in 2021 USD.

Lastly, our analysis includes post-discharge follow-up data in healthcare utilization, clinical outcome, and productivity loss. These data add comprehensiveness to our analysis, in contrast to existing literature that largely focuses on the inpatient period [12, 14, 22]. To this end, our high rate of follow-up also minimizes missing data. Our inclusion of productivity losses and indirect costs of healthcare (e.g. transportation and purchased meals while in hospital) even after discharge is important, for as mentioned, the economic burden of illness on families is not limited to the direct costs of healthcare such as hospital fees and medication costs [24, 34].

### Limitations of analysis

An important limitation of this analysis is that we did not achieve an improvement in the primary target outcome – proportion of children receiving a timely sepsis bundle. Instead, the cost effectiveness observed was driven by other factors, in particular a reduction in admission rate, that may have been confounded by factors such as COVID-related shifts in HRU behavior. The Smart Triage trial was originally designed as a difference in differences study, with a control site able to account for changes unrelated to Smart Triage implementation at JRRH [16]. Unfortunately, delays induced by the pandemic prevented trial initiation at the control site until well after Smart Triage implementation at JRRH, thereby precluding the usability of the control site for this analysis. Reassuringly, in corresponding with local clinicians, no major changes to the triage infrastructure apart from Smart Triage were implemented within the duration of our study. Symptom screening of patients for diversion to COVID designated centers (of which JRRH was not) was implemented in the initial months of the pandemic and encapsulated both phases of the study, thereby less likely to introduce bias. Of course, while these observations are reassuring, the cost-effectiveness of Smart Triage from this analysis requires confirmation in additional sites in a “new normal” era.

Despite potential confounding, a reduction in admission due to Smart Triage is entirely plausible. For example, bringing sicker patients to the front of the assessment queue may have given these patients priority for consideration of hospital admission, a limited resource in LMIC settings. Simultaneously, a low-risk classification by Smart Triage’s data-driven algorithm may have added confidence for clinicians to treat patients with mild to moderate symptoms as outpatients, whereas prior to Smart Triage, a conservative recommendation of admission may have been offered to these patients. While the observed reduction in admissions that drove cost-effectiveness is likely multi-factorial in etiology, our post-hoc sensitivity analysis reassuringly showed that the cost-effectiveness of Smart Triage under the HOC-based threshold is preserved when a penalty factor of up to 50% is applied to the reduction in admission rate.

An additional limitation to our analysis lies in our inability to model years lived with disability due to the lack of long-term quality-of-life data and corresponding disability weights in sepsis survivors in the LMIC context. Extrapolation from HIC data would be inaccurate given the vast differences in social support systems and standards of care between HICs and LMICs. However, our model may be rerun to incorporate such data when available, to calculate incremental cost per DALY averted of Smart Triage. This re-analysis would be informative, as the HOC-based threshold is based on DALYs averted and not YLLs averted. Mathematically speaking, as DALYs are calculated by summing YLLs and years lived with disability, some YLLs averted by Smart Triage may be offset by years lived with disability. This offset would potentially lower the denominator for ICER, increasing the ICER and diluting cost-effectiveness. Nonetheless, our observation of cost-effectiveness well before the HOC-based threshold provides ample room to maintain cost-effectiveness even if years lived with disability were incorporated, despite this potential dilution.

A third limitation lies in our inability to count opportunity costs unrelated to missed wages, such as the death of livestock or spoilage of unattended crops that may have been used to generate income [24]. The variable nature of the source of these losses makes them difficult to count comprehensively, and a review of existing LMIC costing studies show a large variation in the breadth of opportunity costs captured [44]. However, the severity of these opportunity costs is likely linked to higher HRU rates. Therefore, if Smart Triage can indeed reduce HRU such as need for admission, these opportunity costs would likely be reduced.

Finally, scaling up of interventions must consider costs of scale up as well as budget impact [46], which are not captured in a cost-effectiveness analysis at a single site. Nonetheless, the high cost-effectiveness of Smart Triage at JRRH provides ample buffer for unanticipated scale up costs, while the low program cost is reassuring from a budget perspective. A subsequent budget impact analysis, informed by scale up costs measured during a multicenter trial, may be conducted to confirm Smart Triage’s adoptability as an intervention for pediatric sepsis triage at the regional and national levels.

## Conclusion

Smart Triage reached a high probability of cost-effectiveness as defined by an HOC-based cost-effectiveness threshold, which assesses proposed interventions based on the country-specific per dollar impact on healthcare outcomes. This cost-effectiveness provides strong rationale for multicenter scale up. Also, Smart Triage benefits patients both clinically and economically, in terms of YLLs averted and cost savings respectively. Our analysis represents the first application of economic modelling methods to evaluate the cost-effectiveness of a pediatric sepsis triage intervention, providing a more comprehensive analysis than those based on only program costs and surrogate outcomes. Smart Triage’s cost-effectiveness was primarily driven by a reduction in admissions, which may be confounded by changes in healthcare utilization induced by factors such as the COVID pandemic. Reassuringly, sensitivity analyses using a penalty factor of up to 50% for reduction in admissions show persistent cost-effectiveness. Areas for future research include confirmation of our results in a post-pandemic “new normal” era, calculation of cost per DALY averted using long-term disability data when available, and modelling of budget impact based on scale up costs measured through a larger multicenter trial. To this end, we are actively pursuing expansion into additional sites in collaboration with local agencies.

### Supplementary Information

**Additional file 1: Supplementary table 1.** Program costs of Smart Triage in 2021 United States Dollars. Costs marked with * were originally incurred in 2021 Ugandan Shillings and were converted into USD by the average conversion rate in 2021 as per the World Bank (3587.075 Ugandan Shillings to 1 USD). Abbreviations: USD = United States Dollar, JRRH = Jinja Regional Referral Hospital.

**Additional file 2: Supplementary table 2.** Detailed Monte Carlo simulation results from societal, government, and patient perspectives. Costs in 2021 USD. Abbreviations: YLL averted = years of life lost averted, ICER = incremental cost effectiveness ratio, USD = United States dollars.

**Additional file 3:** **Supplementary table 3.** Details of Monte Carlo simulations for sensitivity analyses. Abbreviations: USD = United States dollars, YLL averted = years of life lost averted, ICER = incremental cost-effectiveness ratio.

## Data Availability

The datasets analysed during the current study are available in the Pediatric Sepsis Colab repository, hosted on Borealis, https://borealisdata.ca/dataverse/smart_triage. The corresponding author (EL) may be contacted for data.

## Abbreviations

ARR: Absolute risk reduction
CEAC: Cost effectiveness acceptability curve
CHEERS: Consolidated Health Economic Evaluation Reporting Standards
COVID: Coronavirus disease
DALY: Disability adjusted life year
ETAT: Emergency Triage Assessment and Treatment
GDP: Gross domestic product
HOC: Health opportunity cost
HRU: Health resource utilization
ICER: Incremental cost effectiveness ratio
JRRH: Jinja Regional Referral hospital
LMIC: Lower middle income country
NS: Not significant
RR: Relative risk
UHC: Universal health care
USD: United States dollar
WHO: World Health Organization
YLL: Years of life lost
